# Enhancing Clustering Efficiency in Heterogeneous Wireless Sensor Network Protocols Using the K-Nearest Neighbours Algorithm

**DOI:** 10.3390/s25041029

**Published:** 2025-02-09

**Authors:** Abdulla Juwaied, Lidia Jackowska-Strumillo, Artur Sierszeń

**Affiliations:** Institute of Applied Computer Science, Lodz University of Technology, ul. Stefanowskiego 18, 90-537 Lodz, Poland; lidia.jackowska-strumillo@p.lodz.pl (L.J.-S.); artur.sierszen@p.lodz.pl (A.S.)

**Keywords:** clustering, cluster head position, sensors, LEACH, SEP, TEEN, DEC, KNN, energy consumption

## Abstract

Wireless Sensor Networks are formed by tiny, self-contained, battery-powered computers with radio links that can sense their surroundings for events of interest and store and process the sensed data. Sensor nodes wirelessly communicate with each other to relay information to a central base station. Energy consumption is the most critical parameter in Wireless Sensor Networks (WSNs). Network lifespan is directly influenced by the energy consumption of the sensor nodes. All sensors in the network send and receive data from the base station (BS) using different routing protocols and algorithms. These routing protocols use two main types of clustering: hierarchical clustering and flat clustering. Consequently, effective clustering within Wireless Sensor Network (WSN) protocols is essential for establishing secure connections among nodes, ensuring a stable network lifetime. This paper introduces a novel approach to improve energy efficiency, reduce the length of network connections, and increase network lifetime in heterogeneous Wireless Sensor Networks by employing the K-Nearest Neighbours (KNN) algorithm to optimise node selection and clustering mechanisms for four protocols: Low-Energy Adaptive Clustering Hierarchy (LEACH), Stable Election Protocol (SEP), Threshold-sensitive Energy Efficient sensor Network (TEEN), and Distributed Energy-efficient Clustering (DEC). Simulation results obtained using MATLAB (R2024b) demonstrate the efficacy of the proposed K-Nearest Neighbours algorithm, revealing that the modified protocols achieve shorter distances between cluster heads and nodes, reduced energy consumption, and improved network lifetime compared to the original protocols. The proposed KNN-based approach enhances the network’s operational efficiency and security, offering a robust solution for energy management in WSNs.

## 1. Introduction

**Figure 1:**
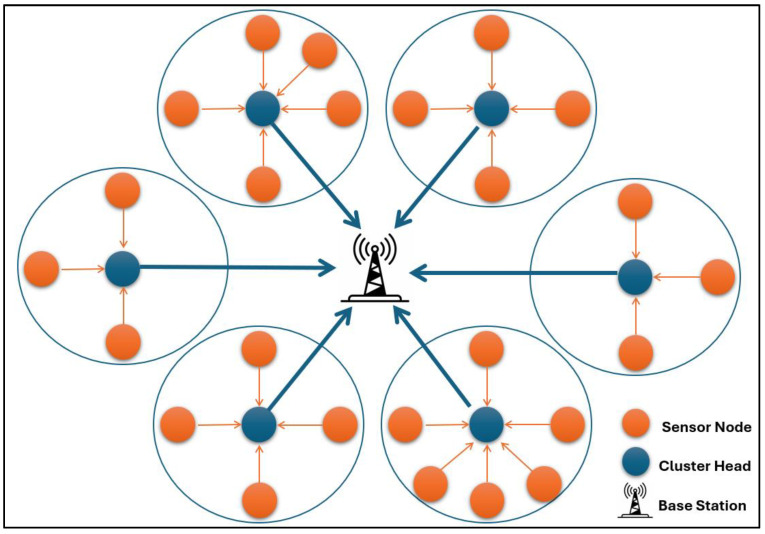
Basic WSN infrastructure.

Wireless Sensor Networks are small, disposable, low-power device sets [1]. They are a new class of ad hoc networks with sensing capabilities. Wireless Sensor Networks (WSNs) have many healthcare, environmental, industrial, and defence applications. Depending on the application, the size of a WSN scales from tens (as in a body area network that monitors the human body) to hundreds (in networks used for industrial monitoring) and thousands (in networks that are used for environmental monitoring and defence applications) of nodes that have sensing, processing, and radio capabilities. In critical areas like defence applications, security is the primary concern. In WSNs, security can refer to protecting the contents of the packets transmitted across the network, authenticating nodes, or protecting the physical locations of the nodes of interest. In the past decade, Wireless Sensor Networks have become an interesting topic for researchers in computer security because the most critical technologies depend on WSNs. All Wireless Sensor Networks consist of small devices called nodes or sensors. These nodes consist of four main units: power, transceiver, processing, and sensing units [1]. First, WSNs were founded for military applications. However, WSNs have become one of the most important approaches for monitoring information technology and are widely used in commercial and industrial areas [2,3,4]. Clustering in Wireless Sensor Network protocols can be divided into flat and hierarchical. The drawback of flat clustering is maintaining large routing tables, which are unsuitable for extensive networks. On the other hand, hierarchical clustering is the second type, which divides the network into small groups of cluster areas. Therefore, most protocols used the second type [3]. Figure 1 shows a standard Wireless Sensor Network model where nodes start to produce data by sensing the environment with sensors in the network. These data will be passed to the sink node in two ways, either through intermediate relay nodes or directly.

These sink nodes will be gateways to the Wireless Sensor Network backbone infrastructure [4]. The backbone infrastructure consists of a user interface to interact with the network, a database to store the data, and local analysis network access tools.

These protocols are developed depending on the basics of the application and architecture of the network. For establishing WSN protocols, several factors should be considered: for example, energy consumption, which is the most important criterion as the network’s lifetime depends on it, and the position of cluster heads in the network. In WSNs, energy efficiency is the key to developing or designing protocols, because the network sensors have a one-time battery backup. Therefore, some protocols depend on extending the lifetime of WSNs by balancing using the battery power of nodes in the network [5]. All Wireless Sensor Networks should have three main properties: high reliability, easy conservation, and easy extension. So, when developing any WSN protocol, these three properties should be considered.

**Figure 2:**
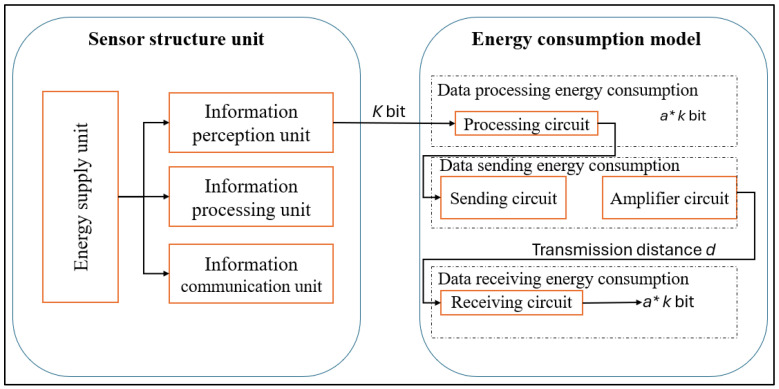
Energy consumption structure of wireless sensor nodes.

Energy consumption is fundamental in designing and implementing a Wireless Sensor Network. Therefore, we can classify energy consumption into three main classes: data sending or sensing, data processing, and data transmission [6]. Figure 2 shows the energy consumption structure of wireless sensor nodes, which consists of three main aspects: data processing energy consumption, data sending energy consumption, and data receiving energy consumption [7,8].

The data processing energy consumption can be calculated from the energy consumption rate of data processing and the amount of data processed, as shown in Equation (1) [9,10]:

(1) $$E_{a}\left(i\right)=i\cdot Ex$$

where *i* is the amount of data processed, and *Ex* is the energy consumption of the data processing circuit for processing unit data.

We calculate the energy consumption of data reception based on the amount of data received, as shown in Equation (2) [9]:

(2) $$E_{b}\left(i\right)=i\cdot Ey$$

where *i* is the amount of data processed as in the previous Equation, and *Ey* is the energy consumption of the transmitting circuit. The energy consumption of the data transmission is mainly related to the amount of data sent and the distance sent, as shown in Equation (3) [9]:

(3) $$E_{Z\;}(i,d)=\left\{\;\;\begin{array}{c} E_{b}\left(i\right)+i⸱E_{m}*\;d^{2},\;(d<d_{0})\\ E_{b}\left(i\right)+i⸱E_{n}\;d^{4},\;(d\geq \;d_{0}) \end{array}\right.$$

where *d* is the distance between two sensors, *d*_0_ is a constant, and the energy dissipation is adopted; the energy consumption of sending data is relative to the square of distance when the distance between sending and receiving nodes is less than *d*_0_ [8,9]. *E_m_* is the energy consumption coefficient of the free space power amplifier. When the distance between the sending and receiving node is greater than or equal to *d*_0_, then the multi-channel model for energy dissipation is adopted. The energy consumption of sending data is relative to the fourth power of distance [9,10]. *E_n_* presents the energy consumption coefficient of multi-channel power amplifiers.

The novelty of this research lies in the innovative integration of KNN machine learning algorithms into WSN protocols to address long-standing challenges in energy efficiency, network reliability, and clustering optimisation. The proposed modifications to existing protocols (LEACH-KNN, SEP-KNN, TEEN-KNN, and DEC-KNN) represent a significant advancement in WSN design by combining the strengths of grouping algorithms with practical network requirements. This study presents new architectures, models, and distributed algorithms that optimise cluster head selection and placement, reduce energy consumption, and extend network lifetime.

## 2. Related Works

Many researchers worked to minimise the energy consumption used in routing. Therefore, different types of protocols have been developed. These protocols can be classified into four main parts: hierarchical, data-centric, location, and network flow protocols [11,12]. In this paper, we will focus on hierarchical protocols. The general idea of routing is to process a specific path in the network from the start point to the endpoint to which the data can be transmitted. Therefore, this paper will focus on four protocols to route the data from node to base station in a Wireless Sensor Network. Some researchers have focused on a methodology that depends on transmitting sensed information between the base station and nodes [13]. Another approach was introduced to save energy, which will be wasted in the cluster head selection in each round, depending on which CH will remain unchanged when it has enough energy [14]. The Ad-LEACH protocol proposed by Qing et al. [15] reduces power dissipation and the complexity of the network based on static clustering. The general idea is to separate the network into small areas to reduce the energy consumption of nodes while transmitting data between the more extensive network. Smaragdakis et al. [16] proposed the SEP protocol in a heterogeneous two-level hierarchical network; each sensor node independently selects itself as a cluster head based on its initial energy compared to other nodes. The Energy Level Based Stable Election Protocol (ELBSEP) [17] was proposed, combining some features of SEP and the energy level estimation method. The proposed method could increase energy efficiency, improving the lifetime of the network. However, ELBSEP is unsuitable for frequent information from Wireless Sensor Networks.

This paper proposes an approach to reduce the number of cluster heads, minimise distances, lower energy consumption, and improve network stability and lifetime. The KNN algorithm was used by the authors to modify the TEEN protocol; this study successfully demonstrates that integrating the KNN algorithm into the TEEN protocol enhances cluster head selection, leading to improved energy efficiency and network performance in WSNs. This modification addresses key challenges in WSN deployment, such as energy conservation and data reliability, making it a valuable contribution to the field [18]. A new approach was used to optimise WSN deployment by combining the KNN algorithm estimation with a modified metaheuristic algorithm, the Intensified-Hitchcock Bird-Inspired Algorithm (I-HBIA), to determine optimal sensor node placement [19]. This study aims to enhance the deployment process by improving decision-making capabilities in order to provide efficient deployment of WSN configurations. The approach will have some drawbacks, such as the increasing number of node dimensions and not accounting for the number of dead CHs and the location of live CHs. A distributed energy-efficient clustering protocol for heterogeneous WSNs is another phase for cluster selection. Regarding the stability period, DCE performs better than the DEEC, SEP, and LEACH protocols [20]. An intrusion detection model based on KNN was proposed in by Juwaied et al.; this study uses a KNN-based intrusion detection system to enhance WSN. It integrates the algorithm for KNN with an arithmetic optimisation algorithm, improving the accuracy and efficiency of intrusion detection. In this respect, the model is simulated for performance testing to prove it detects intrusions within the environment [21]. Another study using a hybrid feature reduction technique was performed; the paper introduces a hybrid model that employs machine learning techniques, including KNN, to enhance the security of WSNs against cyberattacks. The model utilises feature reduction methods like Singular Value Decomposition (SVD) and Principal Component Analysis (PCA) to improve detection accuracy and efficiency [22].

A study [23] used the KNN algorithm to implement a hybrid approach that combines KNN with quantum-behaved particle swarm optimisation to optimise the placement of base stations in WSNs. The proposed method extends the network’s lifetime by minimising energy consumption through optimal base station positioning. KNN is also implemented on mobile WSNs [24]; the study investigates secure KNN query processing in two-tiered mobile WSNs. It proposes a secure KNN query algorithm that protects user privacy and ensures query results’ integrity, addressing communication overhead challenges in WSNs.

One study focused on improving the HEED algorithm for cluster head selection in Wireless Sensor Networks (WSNs). It integrated mathematical modelling, statistical analysis, and a deep artificial neural network (ANN) to address energy consumption and network lifetime inefficiencies, particularly in non-uniformly distributed WSNs. HEED’s probabilistic approach to CH selection can result in suboptimal cluster head placement, especially in heterogeneous networks with varying node energy levels. The iterative nature of HEED’s CH selection process increases communication overhead, which can lead to faster energy depletion in resource-constrained nodes [25].

HMRP-IWSN was proposed, which combines a Hybrid Optimised Deep Neural Network (HODNN) for intrusion detection and a Modified Energy-Efficient Centralised Clustering Routing Protocol (MEECRP) for secure and energy-efficient routing in IoT-enabled WSNs, but it does not take in the consideration the location of CHs in the network. HMRP-IWSN is more appropriate for IoT-enabled WSNs that require energy efficiency and robust security [26].

An Enhanced Intrusion Detection Model Based on Improved KNN in WSNs was presented, focusing on improving Wireless Sensor Networks using the K-Nearest Neighbours algorithm. This method aims to detect intrusion in WSNs, proposing a KNN-based model enhanced with the Arithmetic Optimisation Algorithm (AOA) to detect DoS attacks effectively. It combines KNN with AOA and Lévy flight strategies to optimise intrusion detection accuracy and reduce false positives. This work focused only on security and intrusion detection, ignoring energy efficiency, dead sensor nodes, and network longevity [27].

Iqbal [28] highlighted the importance of multi-objective optimisation in WSNs, emphasising the need to balance trade-offs between energy efficiency, latency, and reliability. Their work explores the use of Pareto optimisation and evolutionary algorithms to achieve these trade-offs.

The work by Arbelaez [29] demonstrates the effectiveness of constraint-based optimisation in solving distance-constrained problems, which is highly relevant to WSN clustering and routing. Similarly, meta-heuristic algorithms can optimise cluster head selection, routing paths, and energy management in WSNs by exploring large search spaces and adapting to real-time changes.

Some machine learning approaches have been proposed to modify WSN protocols, such as Support Vector Machines (SVMs), which effectively handle non-linear data through kernel functions. They have strong generalisation capabilities, making them suitable for complex WSN topologies. SVMs can be used for cluster head (CH) selection by classifying nodes based on energy levels, distance to the base station, and other features. SVMs may outperform KNN in scenarios with non-linear relationships between features, but could have higher computational overhead [30].

Decision trees are other algorithms that are simple to implement and interpret. Their low computational requirements make them suitable for resource-constrained Wireless Sensor Network nodes. This method can be used for hierarchical clustering by splitting nodes based on energy levels and proximity to CHs. Decision trees may provide faster execution than KNN, but could be less accurate in dynamic environments [31].

Biologically inspired algorithms have also been implemented in WSNs. Genetic Algorithms (GAs) are a global optimisation technique that can find near-optimal CH selection and routing solutions. They are adaptable to dynamic network conditions. This algorithm can optimise CH placement and routing paths to minimise energy consumption and maximise network lifetime. GAs may outperform KNN in large-scale networks with complex topologies, but could require more computational resources [32].

Ant Colony Optimisation (ACO) is well-suited for path optimisation and can adapt to dynamic network changes. Its distributed nature aligns with the architecture of WSNs. It can optimise routes to reduce energy consumption and improve data delivery reliability. ACO may outperform KNN in routing optimisation, but could have higher computational complexity [33].

Particle Swarm Optimisation (PSO) has a fast convergence rate and effectively solves continuous optimisation problems. It is simple to implement and computationally efficient. PSO can optimise CH selection and routing paths by considering energy levels, distance, and network density. It also may achieve better energy efficiency than KNN, but could be less interpretable [34].

In our previous work, we used the KNN algorithm to modify the TEEN protocol [18]. This article presents a new modification method by integrating the machine learning KNN algorithm with four WSN protocols: LEACH, SEP, TEEN, and DEC. These protocols have one important common factor: they all deal with energy usage. Therefore, this paper aims to improve the protocol’s efficiency and reliability, making it more robust in handling various network scenarios and improving its performance in real-world applications. There will be no overlapping of CHs in the network, and all cluster regions in the network contain CH.

Our work is mainly focused on static WSN configurations. While dynamic network configurations are essential for specific mobility applications, such as vehicular networks or mobile healthcare systems, static sensor networks remain highly relevant and practical for many real-world applications. Static sensor networks are widely used in security systems, such as surveillance systems with cameras and motion detectors, where stationary sensors continuously monitor specific areas. Similarly, in smart homes and gardens, static sensors monitor and control systems like lighting, temperature, and irrigation. Additionally, meteorological measurements rely on static sensor networks to collect long-term environmental data for weather forecasting and research, such as temperature, humidity, and precipitation. Other applications include industrial monitoring, where static sensors detect equipment faults, and infrastructure monitoring, where they assess the structural health of bridges and buildings. These examples demonstrate the critical role of static sensor networks in various domains, justifying this study’s focus on static configurations.

## 3. WSN Protocols

As mentioned previously, WSN are multiple network devices that communicate with each other while signalling to send and receive information. The protocol structure consists of three main parts: location-based routing, hierarchical network routing, and flat network routing [35].

- Location-based routing

In this routing protocol, each node in the network is identified by its location. Location-based routing can be determined by calculating the distance to the closest neighbouring node to ensure the accuracy of the distance. That is conducted using two techniques: the first is to find the coordinate of the neighbouring node, and the second is to use a global position system (GPS) [36,37,38]. In all routing protocols, energy is the primary factor to be considered. Therefore, the architectural aspect of location-based routing is that all nodes should change their state from active to sleep mode when there is no activity [37].

- Heretical network routing

The general idea of this approach is to cluster all the nodes in the network and then choose cluster heads responsible for sending the information to the sink, which saves communication energy and makes Wireless Sensor Networks more scalable [36]. These protocols work depending on two layers: in the first layer, the CH is selected in the network, and in the second layer, routing is performed [37,38].

- Flat network routing

In flat network routing, all the nodes in the network have the same rules and responsibilities for collecting the data or communicating with the base station. The nodes work similarly [37,39], and the base station sends signals to some cluster areas and waits for a response from the nodes located in these clusters to facility-centric characteristics [26,27,28].

In WSN, feature scaling is a critical preprocessing step in implementing machine learning algorithms, particularly in scenarios involving heterogeneous sensor parameters such as energy levels and spatial positions. Whereas the KNN algorithm is employed for clustering and cluster head (CH) selection, feature scaling ensures fair and accurate distance comparisons between nodes. Feature scaling in WSNs includes heterogeneous parameters, fair distance comparisons, and improved clustering efficiency.

WSN nodes are characterised by diverse parameters, such as spatial positions (coordinates) and energy levels. Spatial positions are typically measured in meters, while energy levels are expressed in joules or nanojoules. Feature scaling ensures that all parameters contribute equally to the distance metric. The method treats each parameter equally by normalising or standardising the data, leading to more balanced and accurate clustering decisions. Without proper scaling, the disparity in the range of values for different parameters can lead to biased results, negatively impacting the clustering process and overall network performance.

As was mentioned previously, WSN can be defined as a collection of a large number of nodes that are used to sense the environment of the network and are able to communicate between them. This section will analyse the communication and implementation of four types of protocols. Then, the modified version for LEACH, SEP, TEEN, and DEC will be presented and compared with the original protocol.

### 3.1. LEACH (Low Energy Adaptive Clustering Hierarchy)

The Low Energy Adaptive Clustering Hierarchy (LEACH) protocol is one of the primary routing protocols for Wireless Sensor Networks. The LEACH protocol is a Time Division Multiple Access (TDMA)-based Media Access Control (MAC) protocol. The main idea of this protocol is to expand the energy or battery lifetime of WSNs through a distribution of the energy load among the sensors or nodes in the network [3,10].

The network will be divided into several rounds using the LEACH protocol. Each round contains two stages: the set-up phase and the steady phase. In the set-up phase, all nodes in the network randomly generate the number 0 or 1. One of these nodes in every cluster area will be chosen as cluster head (CH) depending on the threshold *T*($s_{i}$) value. This value is calculated using Equation (4) [3,4,10].

(4) $$T(s_{i})=\left\{\begin{array}{c} \frac{P_{i}}{1-P_{i}(r\;mod\;\frac{1}{P_{i}})\;},\;\;\;\;if\;s_{i}\in G\\ \;\;\;\;\;\;\;\;\;\;\;\;\;\;\;\;0\;\;\;\;\;\;\;\;\;\;\;,\;\;otherwise \end{array}\right.$$

where *T(s_i_)* is the threshold, *P_i_* is the probability of change of node to become a CH, *r* is the current round number, and *G* is the set of nodes that will be chosen as CHs at each round *r*.

If the random number is less than threshold T(n), the node will be the cluster head of the current round *r*. Then, these nodes in the network act as a CH and send short messages to all other remaining sensors. The nodes will choose to join these regions, called cluster areas, depending on the signal strength of the message sent by the cluster head. Then, every CH will create Time Division Multiple Access (TDMA) schedules according to the number of nodes in the cluster because TDMA puts reminder nodes in sleep mode and protects nodes from network data collision [4]. The last step in this phase is that all nodes belonging to the cluster area will send a message to the CH, which will then be sent towards the base station. In the steady state phase, all nodes in each region will send the collected data in their allotted TDMA slots to CH. Then, every CH in each cluster area will transmit these data in a compressed format to the network’s base station.

**Figure 3:**
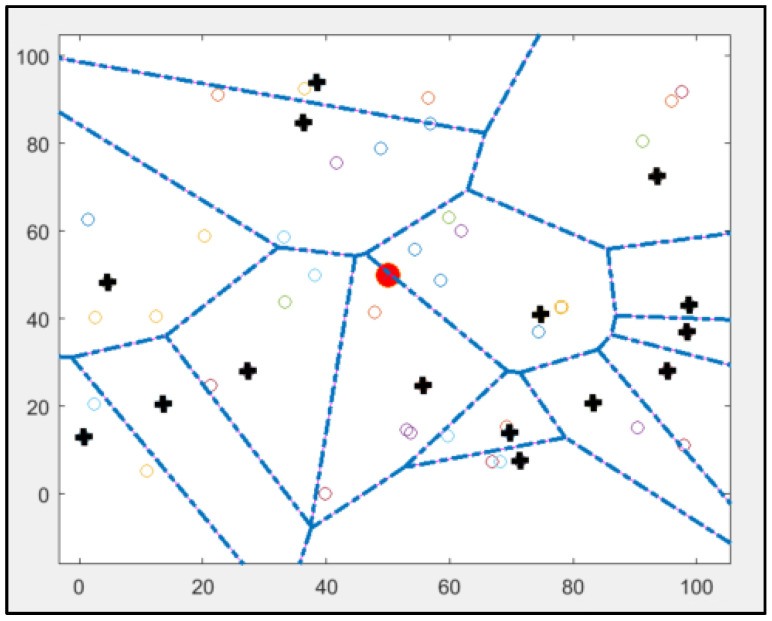
Results of implementing standard LEACH protocol using MATLAB: cluster heads are marked as bold plus signs, other nodes are marked as coloured rhombuses, and the base station is marked as a red circle in the centre.

Figure 3 shows the implementation of the standard LEACH protocol using MATLAB. A standard LEACH protocol was implemented using MATLAB simulation; this figure shows how this protocol needs to provide more explicit information about the position of CHs because it depends only on the probability model. Therefore, some of the CHs are at the edge of the network. Also, some CHs are nearby, and some nodes are not connected to any CH [10,14]. These are the main drawbacks of this protocol: the modified version will not have these drawbacks, and all nodes in the network will choose the nearest CH in the network. As mentioned, all nodes in this protocol will organise themselves in local clusters, with one node acting as cluster head, and run a demised rotation of CH to balance energy consumption in the network.

As seen in the previous figure, some nodes might not be close to the cluster head; therefore, the energy level utilised by the farther sensor does not equal the amount of energy the nearest node uses. These CHs will forward the data to the base station in compressed format. Not all CHs are close to the base station, so they pass these compressed data to neighbouring CHs, which is a multi-hop routing network. 

### 3.2. SEP (Stable Election Protocol)

The stable election protocol is another hierarchical routing protocol for maintaining power consumption in Wireless Sensor Networks. SEP provides stability in the network, but the network’s lifetime decreases after the death of the first sensor in the network. This protocol has two types of nodes: advanced nodes and normal nodes. Therefore, this protocol works in two levels of heterogeneity [33,34].

The threshold is calculated from Equation (4), which is used for each node *si* in each round to check if this node is a cluster head. Because this protocol has two types of nodes, the algorithm of this protocol uses two types of Equations to calculate the probability of nodes. Equation (5) calculates the probability of normal nodes *P_normal_* that will be chosen as CHs in the network [11].

(5) $$P_{normal}=\frac{P_{opt}}{1+m\cdot a}$$

where *P_opt_* is the optimal probability of each node to be chosen as CHs, and *m* is a fraction of total *n* nodes provided with additional energy factor a. On the other hand, Equation (6) is used to calculate the probability of advanced node *P_advanced_* that will be chosen as CHs:

(6) $$P_{advanced}=\frac{P_{opt}}{1+m\cdot a}\left(1+a\right)$$

**Figure 4:**
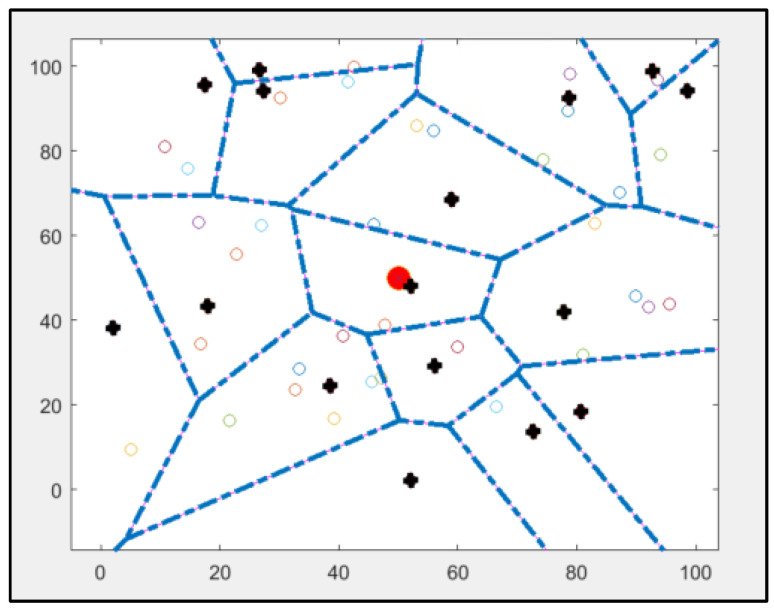
Results of implementing a standard SEP protocol using MATLAB: cluster heads are marked as bold plus signs, other nodes are marked as coloured rhombuses, and the base station is marked as a red circle in the centre.

Because the selection of cluster heads is not dynamic, some sensors that will not be chosen as cluster heads have a high consumption level [33,34]. Therefore, these CHs will die first, which is a drawback of this protocol [40,41]. Figure 4 shows the implementation of the standard SEP protocol using MATLAB.

### 3.3. TEEN (Threshold-Sensitive Energy Efficient Sensor Network)

The Threshold-sensitive Energy Efficient sensor Network (TEEN) protocol employs a hierarchical clustering approach to optimise energy efficiency and data transmission in Wireless Sensor Networks [42]. This protocol utilises a data-centric methodology combined with a two-level hierarchical clustering scheme. At the first level, multiple sensor nodes are grouped into clusters, and one node from each cluster is selected as the CH. At the second level, the CHs from the first level form a higher-level cluster; one of these CHs is designated as the second-level CH. The second-level CH aggregates data from the first-level CHs and transmits them to the base station.

**Figure 5:**
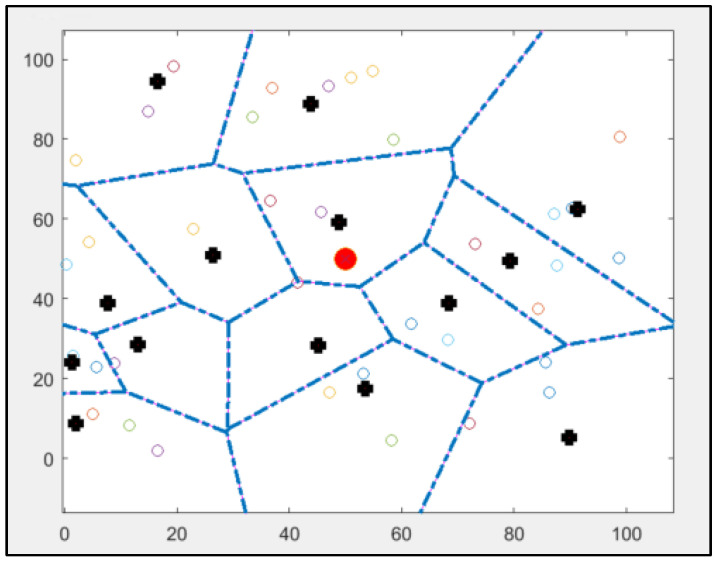
Results of implementing the standard TEEN protocol using MATLAB: cluster heads are marked as bold plus signs, other nodes are marked as coloured rhombuses, and the base station is marked as a red circle in the centre.

Due to its hierarchical clustering mechanism, as illustrated in Figure 5, the TEEN protocol is classified as a reactive protocol [18,43,44]. In this protocol, the CHs within each cluster collect data from their respective cluster members. Once the CHs are selected, the nodes within each cluster begin sensing and transmitting data to their respective CHs, which then broadcast the aggregated data to other cluster areas or the base station [45].

The TEEN protocol differs significantly from protocols such as LEACH and SEP in terms of functionality. Specifically, TEEN introduces two thresholds—Hard Threshold (HT) and Soft Threshold (ST)—to control data transmission and reduce energy consumption. The HT is defined as a user-specified value representing the range of interest for a sensed attribute. When the sensed attribute exceeds the HT, the sensor node transmits data to the CH during the current round. soft threshold is the minimum change of the sensed attribute value, which triggers the sensor’s transmitter. The soft threshold reduces the data transmission frequency because it allows for little change in the sensed attribute. The value of ST in the network can be changed according to the user. If the ST is smaller, the network will be more accurate and have a balanced power consumption [46]. Two conditions allow nodes to transmit data: the value of the hard threshold should be less than the sensed attribute’s current value, and the soft threshold should be equal to or less than the value of the sensed attribute [45]. Figure 5 shows the implementation of the standard TEEN protocol using MATLAB.

### 3.4. DEC (Deterministic Energy-Efficient Clustering)

The deterministic energy-efficient clustering (DEC) protocol effectively enhances energy efficiency and extends the lifespan of Wireless Sensor Networks. This protocol enhances communication and data transfer by arranging the nodes into clusters. The goal of the DEC protocol is to minimise energy consumption by minimising the data transmission overhead and extending the functional lifetime of sensor nodes that rely on battery power [34,46]. The DEC protocol divides the network activities into several rounds, taking two phases: the set-up and steady-state phases. The set-up phase of the protocol involves three key activities: selecting the cluster head, choosing cluster heads randomly, and establishing [47].

**Figure 6:**
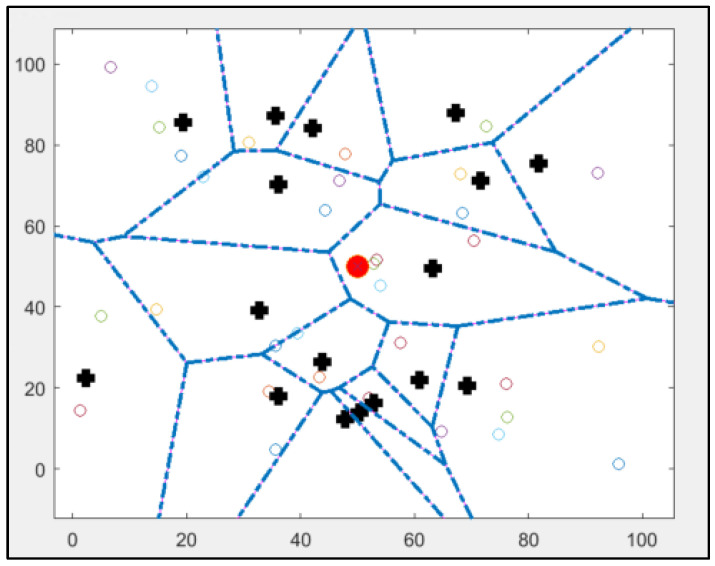
Results of implementing the standard DEC protocol using MATLAB: cluster heads are marked as bold plus signs, other nodes are marked as coloured rhombuses, and the base station is marked as a red circle in the centre.

In the steady-state stage, data transmission will be completed, and the base station will communicate with all cluster heads in the network. This protocol’s set-up stage structure differs from that of other protocols because this stage is categorised into three primary components, as mentioned. However, as the number of rounds in the DEC protocol increases, certain limitations hinder the network’s overall efficiency [47]. For example, it can be seen that the nodes become inactive sooner than they did in LEACH, SEP, and TEEN. Therefore, finding a balance between the stability of the network and the number of active rounds is a significant challenge [47]. Figure 6 shows the implementation of a standard DEC protocol using MATLAB.

### 3.5. Assessment of the Characteristics of the Protocols Under Consideration

Regarding the simulations of the original protocols, we noticed that many limitations appeared. LEACH cluster heads are selected randomly, which can result in uneven energy distribution. Some cluster heads may be located far from the base station (BS), leading to higher energy consumption for data transmission. Some CHs may have no connected nodes, while others may have too many, causing imbalanced energy usage. Cluster heads overlap in coverage, leading to redundant data transmission and wasted energy. LEACH struggles with large-scale networks due to its reliance on random CH selection and single-hop communication. SEP introduces advanced and normal nodes but does not dynamically adjust CH selection based on real-time energy levels, leading to the premature death of high-energy nodes. Cluster heads are selected based on initial energy levels, which can result in inefficient clustering as the network evolves. Some CHs may die early, leaving their clusters disconnected and reducing network reliability. TEEN relies on hard and soft thresholds for data transmission, which can result in data loss if thresholds are not met. Cluster heads may be located at the network’s edges or far from the BS, increasing energy consumption and reducing communication reliability. TEEN is designed for event-driven networks and is unsuitable for periodic data transmission applications. DEC uses a deterministic approach for CH selection, which does not adapt to node energy levels or network topology changes. Nodes with high energy consumption will die early, reducing the network’s longevity. The limitations of the existing protocols (LEACH, SEP, TEEN, and DEC) highlight the challenges in energy efficiency, clustering, and network lifetime in Wireless Sensor Networks (WSNs). These limitations justify the need for the proposed KNN-based modifications.

## 4. KNN in Wireless Sensor Networks

K-Nearest Neighbour is a popular classification tools used to test sets in the network. K-Nearest Neighbour is an example of a lazy learner algorithm because it only builds a model using the training set once the data set is performed and generates a model of the data set beforehand. K-Nearest Neighbour is a supervised learning technique that classifies new query results based on K-Nearest Neighbour groups [36]. The KNN approach is used in numerous areas: artificial intelligence, pattern recognition, statistical estimation, feature selection, and classification challenges. The advantages of this algorithm are its speed of implementation and simplicity.

**Figure 7:**
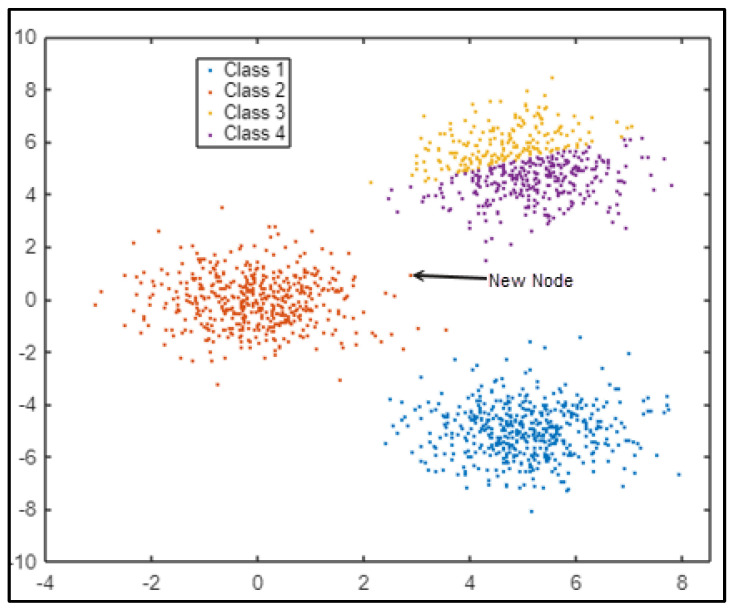
Classification of nodes in the KNN algorithm.

KNN performs well with substantial training data sets and is unaffected by noise. An example of the node classification is shown in Figure 7. The algorithm is looking for one node on a grid to choose if the node is in class 1, 2, 3, or 4. The KNN algorithm attempts to predict which class the new node should belong to. The new node should join class 1 rather than other classes.

K-Nearest Neighbour is a supervised learning algorithm where the results are classified depending on most K-Nearest Neighbour categories. The main idea of this method is to classify a new object depending on attributes and training samples. The algorithm uses a short distance from the query instance to the training samples to determine K-Nearest Neighbours [13] and to calculate the distance between two nodes *D*(*x,y*), where x,y are coordinates of a set of nodes, such as *x* = {*x*_1_, *x*_2_, …… *x_n_*}, *y* = {*y*_1_, *y*_2_, …… *y_n_*}.

This distance between two nodes can be calculated according to one of the following popular distance measures:

- Euclidean distance function: Calculate the distance between binary vectors.

(7) $$D_{\left(x,y\right)}=\sqrt[{2}]{\sum_{i=1}^{K}x_{i}^{2}}-y_{i}^{2}$$

- Absolute distance function: Calculate the distance between the real vectors depending on the sum of their absolute difference.

(8) $$D_{\left(x,y\right)}=\sum_{i=1}^{K}\left|x_{i}-y_{i}\right|$$

- Minkowski distance function: Generalise Euclidean distance functions from Equation (7) and absolute distance functions from Equation (8).

(9) D(x,y)=∑i=1K(|xi−yi|)q1q

When K = 1, it is assigned to the class of its nearest neighbour. The distance between two nodes or sensors depends on intervals. Therefore, the distance result should be based on the arithmetic mean across dataset 0 and standard deviation 1. This can be achieved using Equation (10) by replacing x,y with x′,y′:

(10) $$x^{\prime }=\frac{x-\bar{x}}{Sd\;(x)}$$

where *x* is an unscaled value, the arithmetic mean of which can be calculated from Equation (11), *Sd*(*x*) is a standard deviation which can be calculated from Equation (12), and the result of the scaled value is *x*′.

(11) $$\bar{x}=\frac{1}{K}\sum_{I=1}^{K}x_{i}$$

(12) $$Sd(x)=\sqrt{\frac{1}{K}\sum_{I=1}^{K}{(x}_{i\;}}-\bar{x}{)}^{2}$$

The most popular distance measure to check which of the K instances in the dataset are most similar to a distance measure is the *Euclidean distance function*, which is calculated as the square root of the sum of the squared differences between a new point *x* and existing point *y*:

(13) $$D_{\left(x,y\right)}=\sqrt{{(x}_{1}-y_{1}{)}^{2}+{(x}_{2}-y_{2}{)}^{2}}$$

This type of distance measure is used when the input variable is similar in type; however, if we have different variables, absolute distance functions are recommended. The proposed approach uses Euclidean distance as the primary metric for clustering and cluster head (CH) selection in the Wireless Sensor Network (WSN). While Euclidean distance is a widely used and straightforward metric, it could be a more efficient or effective choice depending on the network topology, node distribution, and application requirements. The WSN nodes are randomly distributed in a 2D plane, and Euclidean distance accurately reflects the actual physical distance between nodes and CHs. The KNN-based clustering approach relies on accurate distance measurements to assign nodes to the nearest CH. Euclidean distance ensures optimal clustering, as demonstrated by the simulation results’ reduced energy consumption and improved network lifetime. Euclidean distance is simple to compute and widely supported in clustering algorithms like KNN. Its computational cost is manageable in the study context, where calculations are performed during the clustering phase rather than on individual nodes.

## 5. Proposed Approach

Energy efficiency is a critical consideration in Wireless Sensor Networks, mainly when the nodes are battery-operated, which is typically the case. Each sensor node is equipped with a finite energy source, and in scenarios requiring smaller sensor nodes, the corresponding batteries must also be reduced in size. This limitation creates significant problems in managing the basic functions of the sensor, such as sensing, communication, and on-board data processing. The energy needed for the transmission of data packets increases with the transmission distance, which requires careful selection of an optimal transmission range to save energy and extend the operational lifetime of the network. In addition, energy consumption levels can increase due to high communication overhead, especially in networks with heavy data traffic. CHs are chosen randomly, and without mechanisms for effective load-balancing, energy depletion often happens unevenly, with particular nodes experiencing much higher energy exhaustion than others.

KNN clustering is a type of supervised machine learning used to segment data into clusters, such that the data points within a single cluster are more similar compared to other clusters. That is particularly advantageous for analysing large and complex datasets, enabling the identification of patterns and trends that would otherwise remain undetected. Clustering also facilitates the prediction of unlabelled data. To enhance the clustering mechanism of WSN protocols, the proposed approach integrates the K-Nearest Neighbours algorithm into WSN protocols. It analyses training data and classifies new test data based on predefined distance metrics. The algorithm identifies the K-Nearest Neighbours of the test data, and the majority of class labels among these neighbours determine classification.

The choice of K in the KNN algorithm is a critical factor influencing clustering efficiency and network performance. A small value of K (e.g., *K* = 1 or 2) can make the algorithm overly sensitive to noise, leading to unstable cluster head selection and uneven energy distribution. Conversely, a large value of K (e.g., *K* > 10) may result in overly generalised clustering, increasing communication distances and energy consumption. To determine the optimal value of K, we conducted simulations with different values of K (*K* = 1, 3, 5, 7, 10) and evaluated their impact on clustering accuracy, energy consumption, and network lifetime. The results indicate that a value of K = 5 provides the best trade-off between clustering accuracy and energy efficiency for the given network parameters. Future work will explore adaptive methods for dynamically tuning K based on real-time network conditions; therefore, the choice of K (number of neighbours) is particularly important, as a small K can make the algorithm overly sensitive to noise, while a large K may reduce clustering accuracy.

The following modification is proposed for the protocols: LEACH, SEP, TEEN, and DEC:

**STAGE 1**: Initialisation

- Network Setup: The network is initialised with n sensor nodes randomly distributed in a 2D field (100 m × 100 m). Each node is assigned initial energy and coordinates (*x, y*).
- Cluster Area and CH Initialisation: Initial CHs are selected using the original protocol’s mechanism All nodes’ coordinates, energy levels, and distances are stored in a database (*DB*).
- Check and Remove Dead Nodes: Check and remove all dead cluster heads and nodes and modify *(DB).*

**STAGE 2**: Distance and energy calculations

- Check Distances:
  ○ Calculate the distance from each CH to the base station.
  ○ Calculate the *TOTAL* distance (*D_total_)* for every CH.
  ○ Calculate the *TOTAL* energy (*E_total_*) for every CH.

**STAGE 3**: Choosing CHs depending on energy consumption and distance to the base station

- Decision Making: Use conditional logic to choose CHs based on *D_total_* and *E_total_*:

**if**  CH_a_D_total_ < CH_b_D_total_ && CH_a_E_total_ > CH_b_E_total_
  Choose CH_a_ as the cluster head in this cluster area and make CH_b_ the normal node in the network.
**Elseif** CH_a_D_total_ > CH_b_ *D_total_* && CHx_a_ *E_total_ *> CH_b_ *E_total_*
  Choose CH_a_ as the cluster head in this cluster area and make CH_b_ the normal node in the network.
**Elseif** CH_a_D_total_ < CH_b_ *D_total_* && CHx_a_ *E_total_ *< CH_b_ *E_total_*
  Choose CH_b_ as the cluster head in this cluster area and make CH_a_ the normal node in the network.
**Else**
  Choose CH_b_ as the cluster head in this cluster area and make CH_a_ the normal node in the network.

- Store Modifications: save new modifications in *DB*_2_

**STAGE 4**: The KNN algorithm is applied to classify nodes based on their proximity to CHs

- The following steps are performed in this stage:
  ○ Input Data: All nodes’ coordinates and energy levels are used as input features.
  ○ Nearest Neighbour Search: The K nearest CHs are identified using the Euclidean distance metric for each node.
  ○ CH Assignment: Nodes are assigned to the nearest CH with sufficient residual energy. If a CH’s energy falls below a threshold, it is excluded from the selection process.
  ○ CH Optimisation: If multiple CHs are located in close proximity, the CH with the highest residual energy and shortest distance to the BS is retained, while others are demoted to regular nodes.
- Load *DB2*: Load the modified database:
          Node = DB2(:,1:2); %get data=coordinate(x,y) from set of data "DB2" at (all row & column 1,2)
- Assigned CH coordination:
          CH = [x,y];
- Select Nearest Nodes: Use the KNN function to select nodes nearest to CH.
          [n,d] = knnsearch(CH,newpoint,’k’,N);
          CH_closest = CH(n,:);
          % coordinate of N neighbour points
          % CH_closest:coordinate(x,y) of no neighbour points
          % n: labelling(order) of each neighbour point
          % d:distance from "each neighbour point" to "CH"
- Implement Results: Highlight the nearest neighbour points in a figure.
  Preparing data for the next round:
          C=setdiff(M,CH_closest,’rows’);
          % returns the data in M that is not in CH-closest,
          % with no repetitions. C is in sorted order

**STAGE 5**: Repeat STAGE 4 until completion

- The KNN algorithm is reapplied to reassign nodes to the nearest active CH.
- The network is prepared for the next round.

The proposed algorithm is based on two main concepts: the number of live cluster heads in the network, and the number of nodes that will join the live CHs using the KNN algorithm. The number of live cluster heads significantly impacts the management of nodes in the network because these cluster heads will manage the nodes in the network that decided to join this cluster head. On the other hand, the BS will receive all information from the nearest cluster heads, which will affect the energy level of the network. The KNN algorithm assists network nodes in determining which cluster heads to join. After excluding all dead nodes, each sensor will select and join only one active cluster head. MATLAB simulation was used to modify LEACH, SEP, TEEN, and DEC protocols. The following table shows the parameters used in the simulation of the protocols. These implementation modifications reduce energy consumption, enhance network stability, and balance the distribution of energy resources across the network.

## 6. Implementation and Simulation

This section presents and compares simulations of the original and modified protocols using the KNN algorithm. Table 1 shows that both simulations used the same parameters.

**Figure 8:**
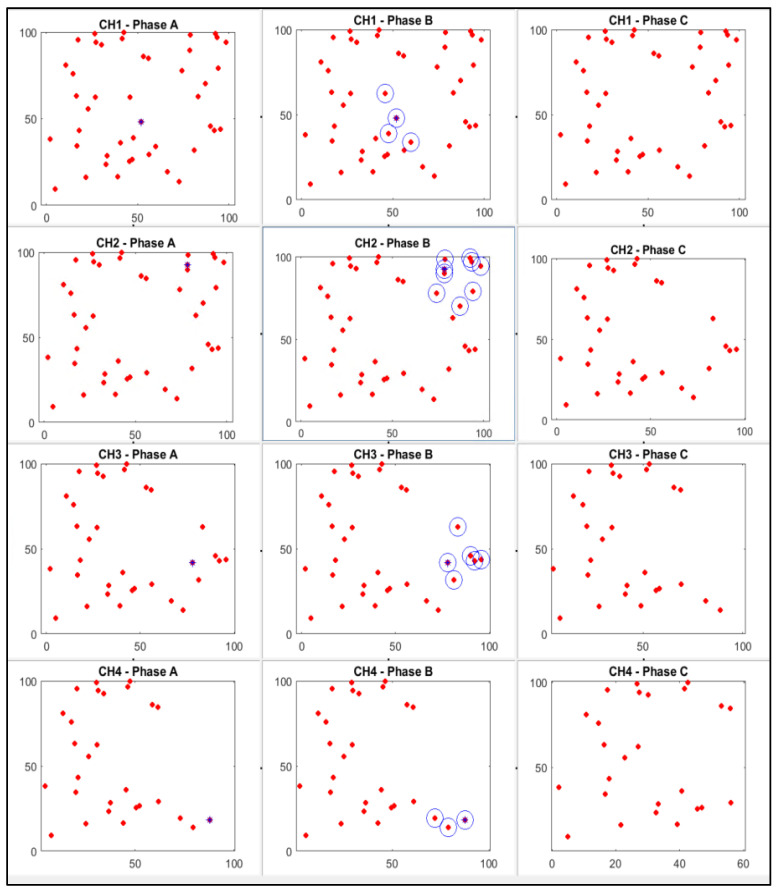
Implementation of CH1–CH4 for LEACH-KNN. Phase A: Assigned coordination of the cluster heads—CH marked with a blue stars, Phase B: Nodes select CH—marked with blue circles around, Phase C: Prepare for next round.

To reduce the network’s energy consumption, all dead nodes in the network will be removed. Some CHs in the network will act as normal nodes after the energy capacity of every CH in the network is checked by calculating the distance between every cluster head to the base station, and the CH energy consumption will be checked to see if it is over the threshold. Figure 8 shows the implementation of the KNN algorithm to improve the clustering of CHs. The clustering is done in the following phases for each cluster head:

- **Phase A:** Implement the network with Table 1 parameters and calculate the initial cluster areas and cluster heads using the original protocol and assigned coordination of the cluster heads.
- **Phase B:** Nodes select nearby CHs using the K-Nearest Neighbours algorithm.
- **Phase C:** Prepare the network for the next round and repeat Phase B (without nodes that selected CH from Phase B).

### 6.1. LEACH-KNN

As was mentioned in Section 2 and shown in Figure 3, the implementation of the original LEACH contained 15 clusters. The network consists of nodes with varying energy consumption levels randomly sorted into 15 groups. Cluster heads, shown by black circles, connect all nodes to the central base station in the network. Although all nodes have identical connection and processing capabilities, their energy consumption varies.

Some nodes, such as cluster heads, use more energy than others in the network. From Figure 3, some drawbacks can be noticed, such as three CHs with no connected nodes and more than two CHs being far away from the centre.

The original LEACH and LEACH-KNN implementations were performed in MATLAB using the same parameters and initial conditions. After implantation, the simulation environment consists of 50 nodes randomly distributed in the 100 m × 100 m field using the same parameters and initial conditions specified in Table 1. The network is heterogeneous, and the node sensors have different energy consumption levels. In the centre of the network is the base station (BS). Figure 8 shows the three-phase simulation using the LEACH-KNN implementation as an example (for CH1, CH2, CH3, and CH4), which was performed using the parameters and initial conditions specified in Table 1.

### 6.2. SEP-KNN

The original implementation of the Stable Election Protocol (SEP) comprises 16 cluster heads, as illustrated in Figure 4, and operates under a framework characterised by two levels of heterogeneity. The selection of cluster heads is conducted randomly, contingent upon the probability assigned to each sensor node. Simulation results of the original protocol indicate that certain cluster heads are positioned at considerable distances from the base station, resulting in increased energy consumption during data transmission between the nodes and the base station. Notably, the original protocol includes two cluster heads that do not serve any associated nodes.

In contrast, the modified approach will retain the same parameters and initial conditions utilised in the initial MATLAB implementation of the original SEP protocol. The simulated network will consist of 50 nodes randomly distributed within a 100 m × 100 m area. The sensor nodes within this heterogeneous network exhibit varying energy consumption profiles, with the base station located at the centre of the network.

### 6.3. TEEN-KNN

The simulation of the original TEEN shows 14 clusters. Conversely, when the energy consumption values are below the threshold, cluster heads cannot send information to the network’s base station. That will be unsuitable for periodic reports applications, because the user may not receive any data. Also, the original protocol shows that the data may be lost if some cluster heads are not in the range of communication (the CH position on the edge of the network or far away from BS).

The original TEEN protocol was implemented using MATLAB simulation on the field with a 100 m × 100 m coordinate size. All CHs in the network that contain energy lower than the threshold or are far away from the base station will act as normal nodes.

### 6.4. DEC-KNN

**Figure 9:**
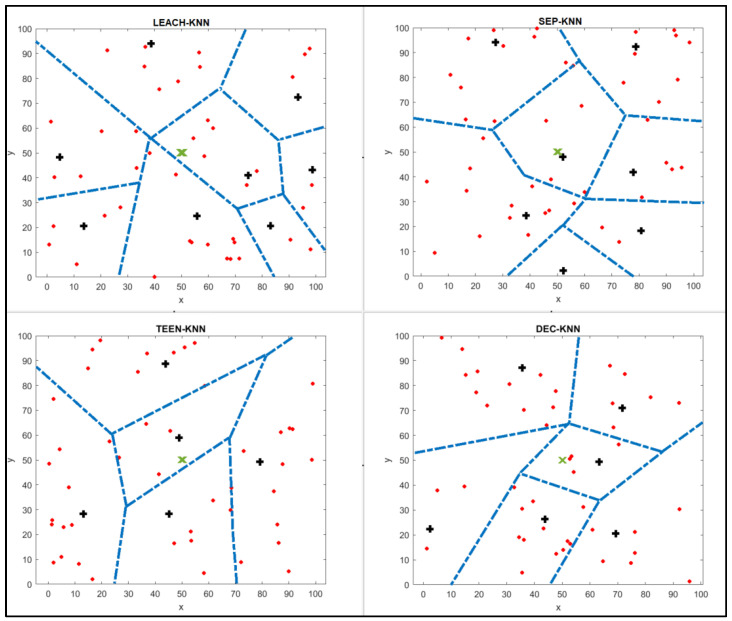
Results of implementing the proposed approaches: LEACH-KNN, SEP-KNN, TEEN-KNN and DEC-KNN. Cluster heads are marked as bold black plus signs, base stations as green x’s, and other nodes as red points.

The results of the DEC-KNN approach are shown in Figure 9. The network will use only six cluster heads to cover the connection of all sensors. All the nodes will choose the nearest CH to be chosen; therefore, the cluster head selection was improved. The dead nodes will not be chosen as CHs in the network. Using the KNN algorithm to choose the best cluster head position for nodes to minimise energy consumption and maximise the network’s lifetime also avoids any CHs overlapping in the same area.

The simulation and analysis for the original DEC and DEC-KNN protocols were performed using MATLAB for the same parameters and initial conditions. The base station (BS) is always located in the centre of the network. In the original protocol, the network has 17 clusters, each consisting of nodes with varying energy levels that have been randomly sorted. The proposed approach containing CHs was organised into six clusters. Summation was performed for 50 randomly distributed nodes in the 100 m × 100 m field.

## 7. Simulation Results

Figure 9 shows the results of the modification protocols: LEACH-KNN, SEP-KNN, TEEN-KNN, and DEC-KNN. When comparing the simulation results between original and modified protocols, we notice that the proposed approach performs better in network clustering. The distance between CHs and the base station is shorter than the original protocol, and there is only one cluster with no nodes using the SEP-KNN approach. The network does not contain dead cluster heads, and there are no CHs on the network’s edge.

### 7.1. Clustering Results

**Figure 10:**
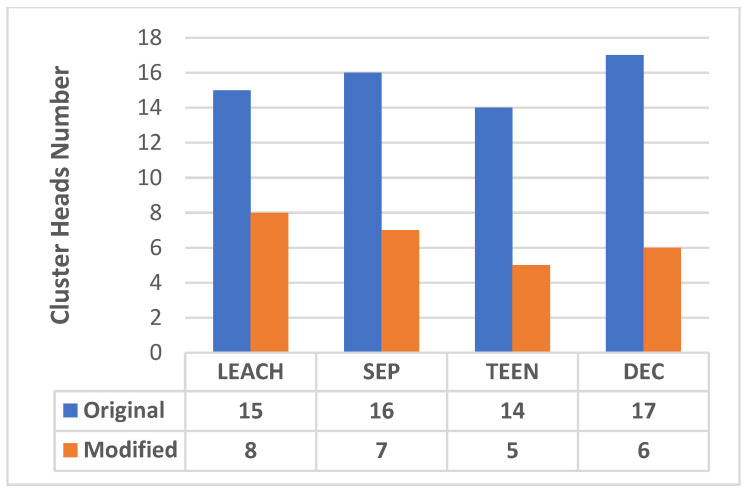
Comparison of total CHs between the original protocol and the modified protocols.

CH number in the original protocol were compared with the modified approaches, as shown in Figure 10. The new approach will divide the network into compact clusters, and most of the CHs will be near the base station, reducing the network’s energy consumption.

**Figure 11:**
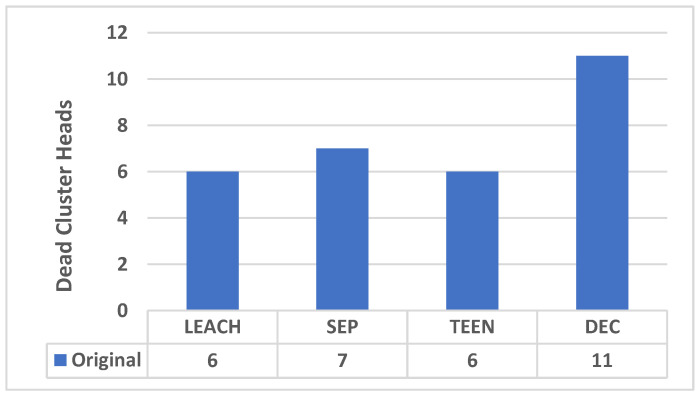
Total dead cluster heads in the original protocols.

In addition, the new approach will not contain dead CHs; on the other hand, Figure 11 shows the number of dead cluster heads in the original protocols. Therefore, the new approach will make the clustering more efficient as no cluster heads will die.

**Figure 12:**
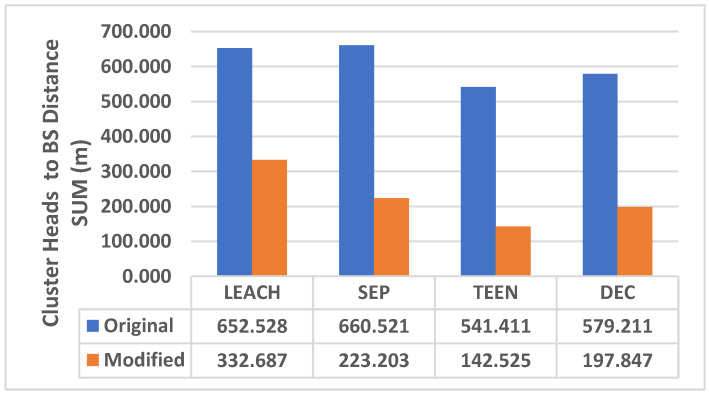
Cluster head distance summation to the base station.

The distance summation of CHs to the base station in the original and modified protocols is shown in Figure 12. The cluster heads will remain in the same place, but the number of joined clusters will be changed depending on the position of the cluster head and energy consumption.

The results in Table 2 show the composition of the longest distance between CHs and the base station in the original and modified protocols. After KNN clustering, nodes will choose CHs nearer to the base station. The proposed method has fewer CHs compared to the original protocol. Therefore, the base station of the network will lose information because of dead cluster heads; the proposed approach focused on all nodes joining the cluster heads with the best location and a high energy level.

### 7.2. Distance Comparison Between CHs

**Figure 13:**
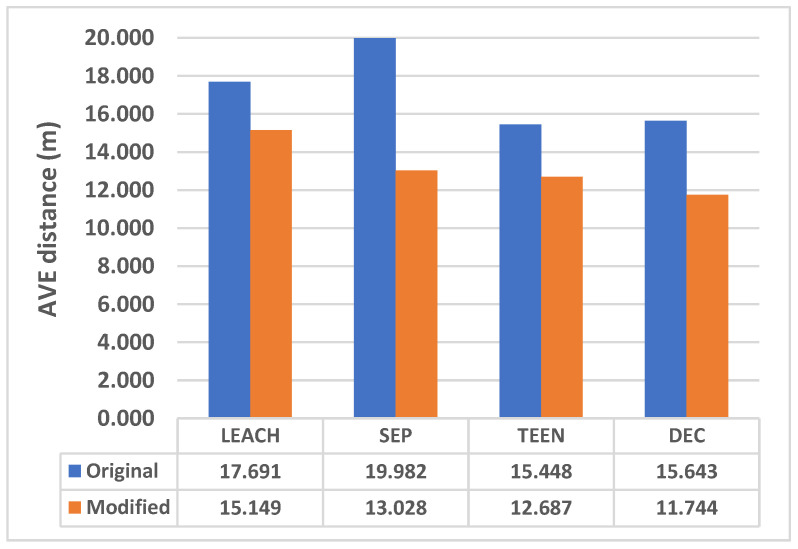
Average distance per node in the original and modified protocols.

A distance comparison for both the original and modified protocols was performed using MATLAB and is shown in Table 3. As shown in Figure 9, the proposed approach will divide the network into more professional clusters. Therefore, the distance summation between nodes in the cluster is less than in the original protocol. The compression of the average distance per node in the original and modified protocols is shown in Figure 13.

### 7.3. Energy Consumption

**Figure 14:**
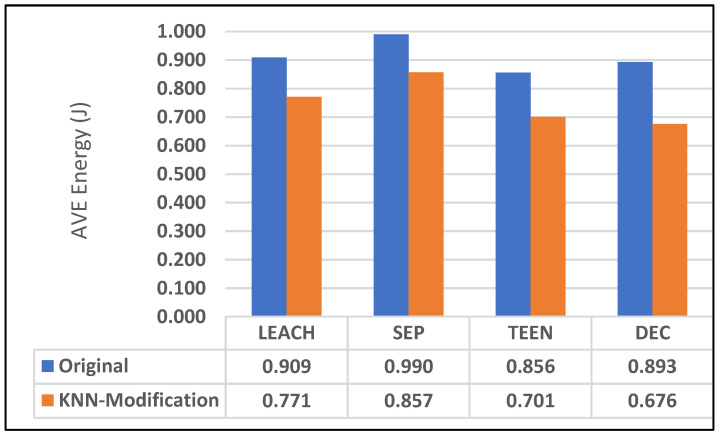
Average energy consumption per node in the original and modified protocols.

Energy usage and dead nodes are significant concerns regarding power management in Wireless Sensor Networks or comparable systems. Dead nodes can affect system performance and conflict with network activities. The average and total distances for the modified approach were lower than those for the original protocol, as mentioned in the previous section. Therefore, CHS’s average energy consumption and the network’s overall energy consumption will be less than the original protocols. Table 4 shows the network’s energy summation, and Figure 14 shows the average energy consumption per node.

### 7.4. Network’s Reliability and Lifetime

**Figure 15:**
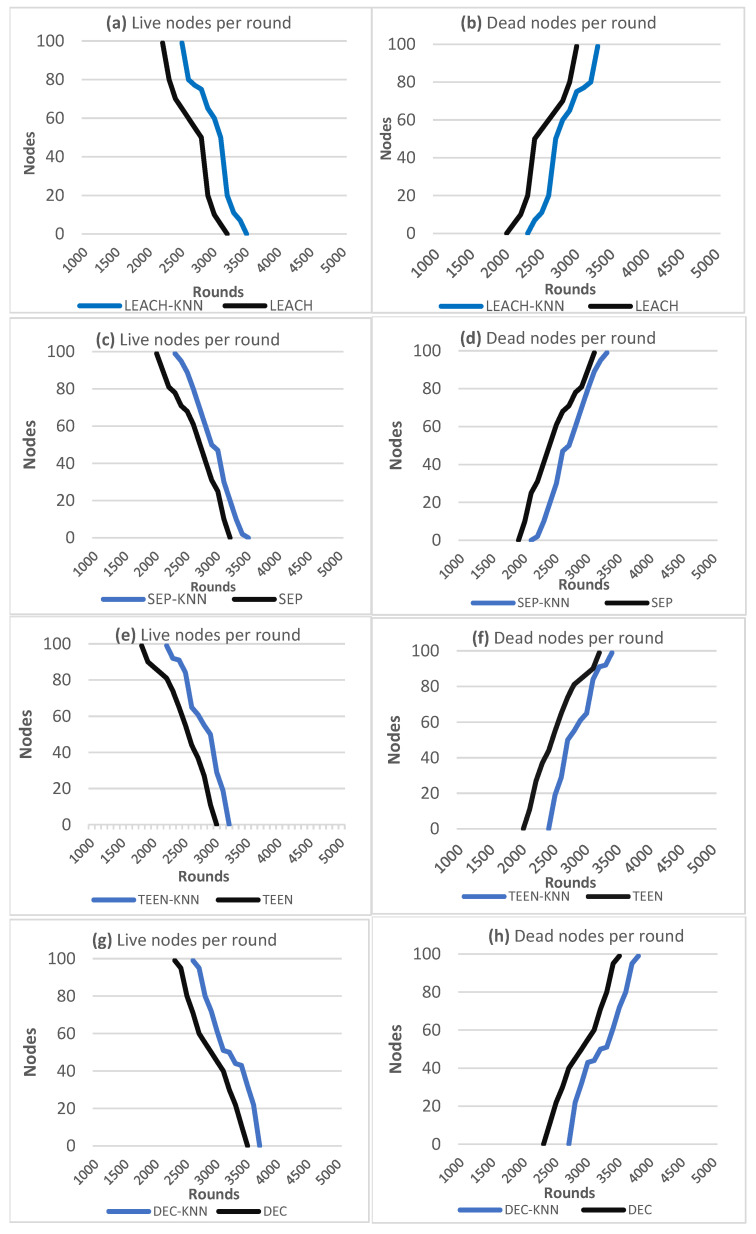
Live nodes and dead nodes per round; (**a**,**b**) LEACH and LEACH-KNN, (**c**,**d**) SEP and SEP-KNN, (**e**,**f**) TEEN and TEEN-KNN, (**g**,**h**) DEC and DEC-KNN.

Computer simulations were used to evaluate the network stability and lifetime across 5000 rounds using values from Table 1 and the base station located in the middle of the network. To check the network stability and lifetime, the following indicators of performance were evaluated: *FSD* is the network’s stability period, which is defined by the number of rounds before the first sensor node dies; *LSD* is the network’s instability period, which is defined by the number of rounds until the final sensor node dies or the period between *FSD* and *LSD*; and *PSA* is the number of rounds until 90% of the sensor nodes remain alive. The charts show the total number of living sensor nodes in a round, including those with energy greater than 0, and show the total number of dead nodes in a round. The simulation results are shown in Figure 15, and Table 5 shows the network’s stability and lifetime performance metrics.

According to the computer simulation and implementation, the proposed algorithm decreased the overall energy consumption by 14.1% in LEACH-KNN, 15.6% in SEP-KNN, 18% in TEEN-KNN, and 22.7% in DEC-KNN. The total distance between CHS was compared between the original and modified protocols; the total distance in the new approach will be decreased by 14.4% in LEACH-KNN, 34.81% in SEP-KNN, 19% in TEEN-KNN, and 25% in DEC-KNN. The proposed algorithm improves network connectivity by reducing link distance between cluster heads and the base station: 49.1% in LEACH-KNN, 66.3% in SEP-KNN, 73.7% in TEEN-KNN, and 65.9% in DEC-KNN. The average distance between CHS and the base station will also be reduced by 4.5% in LEACH-KNN, 2.5% in SEP-KNN, 26.3% in TEEN-KNN, and 3.3% in DEC-KNN. Additionally, the new method significantly increased the network’s lifetime and stability.

## 8. Discussion

Implementing the K-Nearest Neighbours algorithm in Wireless Sensor Network protocols has significantly improved energy efficiency and network lifetime. This study focused on the modification of four protocols: LEACH, SEP, TEEN, and DEC, using the KNN algorithm to optimise cluster head (CH) selection and clustering mechanisms. The results of the MATLAB simulations provide a comprehensive view of the benefits achieved by this approach.

The modified protocols, LEACH-KNN, SEP-KNN, TEEN-KNN, and DEC-KNN, showed a significant reduction in energy consumption compared to their original versions, as follows: the LEACH-KNN reduction is 14.1%, the SEP-KNN reduction is 15.6%, the TEEN-KNN reduction is 18%, and the DEC-KNN reduction is 22.7%. This reduction is attributed to the optimised selection of cluster heads, which minimises the data transmission distance and balances the energy load across the network.

The proposed approach optimises CHs, resulting in a more efficient clustering structure, with fewer cluster heads required to cover the network area. The number of cluster heads was reduced by 46.7% in LEACH-KNN, 56.3% in SEP-KNN, 64.3% in TEEN-KNN, and 64.7% in DEC-KNN. This reduction reduces the energy consumption associated with maintaining multiple cluster heads and reduces the likelihood of overlapping clusters, which can lead to data redundancy and increased energy consumption.

The sum of the distance between the cluster heads and the base station in the four modified protocols was significantly reduced compared to the original protocols, improving the connectivity of the network and reducing the energy required for data transmission. The average distance between CHs and the base station was reduced by 49.1% in LEACH-KNN, 66.3% in SEP-KNN, 73.7% in TEEN-KNN, and 65.9% in DEC-KNN. The proposed approach significantly increased the stability and lifetime of the network, and the LSD metrics were improved in each modified protocol, representing more extended operation time in both the first and the last nodes when the energy is completely exhausted. With these improvements and modifications, the KNN-based approach effectively extends the operational lifetime of the network, allowing for more extended data collection and transmission.

Although our work is mainly focused on static WSN configurations, integrating the KNN algorithm into WSN protocols (LEACH, SEP, TEEN, DEC) shows that the proposed approach could be effectively adapted to dynamic topologies and varying traffic loads by addressing key energy efficiency, clustering optimisation, and network lifetime challenges. For changing topologies, KNN ensures that CHs are selected based on proximity, energy levels, and distance to the base station, dynamically adapting to node failures or removing dead nodes, and that clustering recalibrates in each round. KNN also prioritises nodes with higher energy for CH roles, balancing energy consumption and extending network lifetime, and efficiently handles networks of varying sizes by dynamically adjusting clusters and CHs. Load balancing in KNN minimises communication distances, and traffic across CHs is evenly distributed to prevent overloading. the threshold-based data transmission in TEEN-KNN uses hard and soft thresholds to reduce unnecessary transmissions, optimising energy use during high or low traffic periods. Efficient data aggregation hierarchical clustering reduces data redundancy and transmission overhead. Proximity-based routing nodes are assigned to the nearest CH, minimising energy consumption and improving response to traffic variations. The KNN-based approach dynamically adapts to changing topologies and traffic loads by optimising CH selection, reducing energy consumption, and improving network stability. It ensures efficient clustering, resulting in fewer dead nodes and extended network lifetime, making it a robust solution for dynamic WSN environments.

For computational overhead, the KNN algorithm requires calculating distances between nodes to identify the nearest neighbours. In larger networks, distance computations increase significantly, as each node must compare itself to multiple neighbours. For a network with *n* nodes, the computational complexity of KNN is O (*n × k*) per node, where *k* is the number of nearest neighbours. This scaling results in higher energy consumption for the processing units of sensor nodes, especially in dense networks with large neighbours. In addition to computation, the algorithm introduces communication overhead, as nodes must exchange information such as location, energy levels, and distances to determine the nearest neighbours. In larger networks, this results in increased data transmission and reception, which are the primary sources of energy consumption in WSNs. However, the initial data exchange for KNN computations can still be energy-intensive, particularly in networks with high node density or large geographical areas. In dense networks, each node has more neighbours, leading to more distance calculations and larger data packets for communication. This increases both computational and communication energy consumption. Conversely, in sparse networks, the distances between nodes are larger, which increases the energy required for long-range communication.

The feasibility of the proposed approach in real-world applications depends on whether the energy savings from clustering outweigh the computational cost of KNN.

This approach optimises clustering, reduces energy consumption, and extends network lifetime by leveraging real-time adjustments based on network density and energy levels. Below is a consolidated explanation of how adaptive parameter tuning works and its benefits.

Dynamic adjustment of k (number of nearest neighbours): A higher K value results in larger clusters, reducing the number of cluster heads (CHs) but increasing intra-cluster communication overhead. A lower K value creates smaller clusters, reducing intra-cluster communication but increasing the number of CHs, which can lead to higher energy consumption for inter-cluster communication.

Dynamic adjustment of distance thresholds: Distance thresholds determine which nodes are eligible to join particular CH. Smaller thresholds ensure that only nearby nodes join a CH, reducing intra-cluster communication energy and potentially increasing CHs.

Benefits of adaptive parameter tuning: The proposed approach reduces energy consumption by dynamically optimising clustering parameters. For example, the modified protocols (LEACH-KNN, SEP-KNN, TEEN-KNN, and DEC-KNN) demonstrated energy reductions of up to 22.7% in simulations.

MODLEACH [13] improves energy efficiency by introducing adaptive thresholds for CH selection. The approach does not void dead CHs and overlapping clusters and focuses on reducing energy dissipation without clear information about improvements in network lifetime. ELBSEP [45] improves energy efficiency by combining SEP with energy-level estimation, but ELBSEP does not consider other factors like node distance to the base station or data transmission requirements, which could further optimise performance. Modified TEEN improves energy efficiency with multi-hop routing [43], but multi-hop and multi-path routing add complexity to the protocol. This requires additional path discovery and maintenance computations, which can increase the processing overhead on resource-constrained sensor nodes. The proposed TEEN-KNN protocol ensures compact clusters and eliminates dead CHs, while the modified TEEN in [43] does not explicitly optimise CH placement. Multitier DEC improves energy efficiency through hierarchical clustering [46], but does not explicitly eliminate overlapping CHs or reduce CH-o-BS distance. None of the works demonstrated a 20% reduction in energy consumption compared to the original protocols, proving the KNN method’s effectiveness.

## 9. Conclusions

This paper introduced a new modification approach using the KNN algorithm. Different types of protocols have been chosen to which to apply the new algorithm. The common aspect among LEACH, SEP, and TEEN is energy consumption. Therefore, a common drawback is that, if the threshold of these protocols is reached, there will be a connection, and the user will not know where the dead nodes or cluster heads are in the network.

Integrating the K-Nearest Neighbour algorithm into Wireless Sensor Network protocols offers more compact and efficient clusters with better CH distribution. These modified protocols considerably improve energy consumption in the network and operational stability by optimising cluster head selection and reducing transmission distances. This paper emphasises reducing power consumption within the network by removing all inactive CHs, resulting in a network configuration that consists solely of active CHs. These results highlight the potential of machine learning techniques, such as KNN, to address critical challenges in WSNs, paving the way for more sustainable and efficient network designs.

In this modified framework, all nodes within the network utilise the K-Nearest Neighbours (KNN) algorithm to select the nearest CHs. The implemented modifications ensure no overlapping CHs within the network, ensuring each cluster area is assigned a single CH. Table 5 compares the results for the modified Low-Energy Adaptive Clustering Hierarchy (LEACH), Stable Election Protocol (SEP), and Threshold-sensitive Energy Efficient sensor Network (TEEN) protocols, highlighting the differences in energy consumption based on the configuration of the CHs. The KNN approach facilitates using fewer CHs to cover the network area effectively.

Although the primary focuses of our study were energy efficiency, distance management, and network lifetime, the proposed modifications inherently contribute to other objectives. The proposed approach reduces the communication overhead and transmission delays by optimising the selection of cluster heads (CHs) and minimising the distance between nodes and CHs. This indirectly improves latency, as shorter distances result in faster data transmission. The KNN algorithm ensures that CHs are selected based on their residual energy and proximity to the base station (BS). This prevents premature CH failures and ensures consistent data transmission, thereby enhancing network reliability. Dead nodes and CHs are excluded from the clustering process in each round, ensuring that only active nodes participate in the network. This dynamic adjustment improves the fault tolerance of the network by maintaining connectivity and functionality despite node failures.

After applying the evaluation algorithm to the protocols, less energy consumption appears in the network after removing the dead cluster heads. The new approach improves network lifetime and decreases energy consumption by using a smaller number of CHs to cover all networks and minimise the distance between the CHs and nodes. As was shown in Table 4, the proposed approach shows less energy dissipation, no dead cluster heads, a smaller number of dead nodes, and fewer cluster areas. All cluster areas in the network had CH, and there was no overlapping of CHs in the network. While the current study focuses on simulations using MATLAB, future work will include practical implementations in testbed environments. These environments will simulate dynamic and unpredictable events, such as node failures, environmental interference, and varying energy levels. This will allow us to evaluate the robustness and adaptability of the approach under real-world conditions. Additionally, we plan to incorporate mobility models to simulate dynamic topologies and assess the performance of the proposed protocols in such scenarios. Research could explore the application of other machine learning algorithms to enhance further the adaptability and performance of WSN protocols and real-world testing to validate the simulation results.

## Figures and Tables

**Table 1:** Parameters used to implement the proposed protocols in MATLAB.

Parameters	Definition	Definition
*x* × *y*	100 m × 100 m	Area of network, dimensions
*n*	50	Number of nodes in the network
*R_max_*	5000	Maximum number of rounds
*P_opt_*	0.1	The probability of a node to become a CH
*E_elec_*	50 nJ/bit	Energy dissipation per bit
*E_fs_*	10 pJ/bit/m^2^	Energy dissipation for free space
*E_mp_*	0.0013 pJ/bit/m^4^	Energy dissipation for multipath delay
*E_Rx_*	50 nJ/bit	Receiving energy of sensor
*E_D_*	5 nJ/bit/message	Data aggregation energy
*P_x_*	0.1	Probability of a node to become a cluster head
*L*	4000 bits	Packet size

**Table 2:** Comparison of the longest distance between CH and base station for original and modified protocol.

Protocol Name	X (m)	Y (m)	Distance (m)	Results for Protocols with KNN
LEACH	0.781	13.081	61.526	20% closer to BS
LEACH-KNN	98.796	43.152	49.274	
SEP	98.503	94.137	65.580	21.9% closer to BS
SEP-KNN	78.719	92.470	51.269	
TEEN	1.900	8.722	63.382	32.6% closer to BS
TEEN-KNN	13.077	28.485	42.733	
DEC	19.465	85.605	46.905	15% closer to BS
DEC-KNN	35.572	87.204	39.903	

**Table 3:** Total distances between sensors in the original and modified approaches.

Protocol Name	Original (m)	KNN-Modification (m)	Improvement for Protocols with KNN
LEACH	826.719	757.449	14.4%
SEP	999.091	651.397	34.81%
TEEN	779.909	634.361	19%
DEC	782.129	587.218	25%

**Table 4:** Total energy consumption between sensors in the original and modified approach.

Protocol Name	Original (J)	KNN-Modification (J)	Improvement for Protocols with KNN
LEACH	50.022	42.455	Less 15.1%
SEP	48.534	41.985	Less 13.4%
TEEN	47.677	39.027	Less 18.1%
DEC	44.980	34.005	Less 24.3%

**Table 5:** Performance metrics of the networks’ stabilities and lifetimes.

Protocol	FSD	PSA	LSD	Improvement of the KNN Approach		
				FSD	PSA	LSD
LEACH	2230	2301	3219	12.69%	8.36%	9.25%
LEACH-KNN	2554	2511	3547			
SEP	2065	2107	3217	12.50%	15.31%	8.94%
SEP-KNN	2360	2488	3533			
TEEN	1761	1803	3049	21.35%	23.80%	5.34%
TEEN-KNN	2239	2366	3221			
DEC	2321	2471	3510	12.12%	9.59%	5.82%
DEC-KNN	2641	2733	3727			

## Data Availability

The data is not available because it is used for a PhD thesis in progress.
